# Change Publication

**Published:** 1891-02

**Authors:** 


### EDITORIAL DEPARTMENT.

We beg to inform our readers that the contract with Dr. A. L. Hummel for the publication of THE JOURNAL OF COMPARATIVE MEDICINE AND VETERINARY ARCHIVES having expired this periodical will now be published by W. R. Jenkins, the well-known publishing firm of No. 851 and 853 Sixth Avenue, New York city.

It is requested that all communications be forwarded to the managing editor, Dr. R. S. Huidekoper, corner of Park Avenue and 42d Street, New York city, or in care of W. R. Jenkins. It is the aim of the editors to keep the Journal up to the same high standard as heretofore and also to introduce several new features, one of which will be found in this number giving illustration of the various improvement's in all articles necessary for the care of live stock which have been proctected by our government.

